# Don’t Panic & Other Thoughts on Crystallographic Refereeing

**DOI:** 10.1063/4.0001104

**Published:** 2025-10-27

**Authors:** Christine M Beavers

**Affiliations:** 1 Rigaku Corporation, Akashima, Tokyo, Japan; 2 Rigaku Americas, The Woodlands, Texas, USA

## Abstract

When one embarks on the journey of discovery that is crystallographic reviewing, one should prepare themselves properly; sometimes, not even a towel is enough. No, you may also require dogged persistence, a penchant for investigations, and a nigh-on-masochistic desire for properly modeled disorder. Maybe that last bit is a personal foible; your needs may vary. Please come and hear about the good, the bad and the ugly that has pounced upon me, during my wanderings as a crystallographic referee, mostly in the wilds of small molecule and high- pressure crystallography.

